# The ENTER study (E-DetectioN Tool for Emerging Mental DisoRders): general population recruitment and data integrity in online screening for psychosis risk

**DOI:** 10.3389/fpsyt.2025.1665854

**Published:** 2025-11-18

**Authors:** Phoebe Wallman, Andrés Estradé, Matilda Azis, Kate Haining, Xinyi Liang, Thomas J. Spencer, Kelly Diederen, Umberto Provenzani, Peter J. Uhlhaas, Paolo Fusar-Poli

**Affiliations:** 1 Department of Psychosis Studies, Institute of Psychiatry, Psychology and Neuroscience, King’s College London, London, United Kingdom; 2 School of Psychology and Neuroscience, University of Glasgow, Glasgow, United Kingdom; 3 Outreach and Support in South-London (OASIS) Service, South London and Maudsley (SLaM) NHS Foundation Trust, London, United Kingdom; 4 Department of Brain and Behavioral Sciences, University of Pavia, Pavia, Italy; 5 Department of Psychiatry and Psychotherapy, University Hospital, Ludwig-Maximilian-University (LMU), Munich, Germany; 6 Department of Child and Adolescent Psychiatry, Charité - Universitätsmedizin Berlin, Berlin, Germany

**Keywords:** clinical high risk (CHR) for psychosis, psychosis risk, attenuated psychotic symptoms, online screening, online fraud detection, data quality

## Abstract

**Introduction:**

Effective detection of young people at clinical high-risk for psychosis (CHR-P) is one of the rate-limiting steps in improving outcomes through preventive treatment. ENTER (E-DetectioN Tool for Emerging Mental DisoRders) was developed to refine and increase the specificity of e-detection strategies to identify young people in the community who might be exhibiting emerging symptoms of psychosis. This paper aimed to outline the ENTER procedure and data validation process and the characteristics of the self-selected sample.

**Methods:**

The ENTER study was conducted across sites in London, Glasgow (UK), and Pavia (Italy). Participants from the general population aged 12–35 years were recruited through universities, colleges, flyers, and social media. The online screener collected data on demographics, cognition, speech, environmental risk and protective factors, and frequent subthreshold features that characterise emerging psychotic disorders.

**Results:**

A total of 8,009 participants completed the screener over a period of 3 years. However, only 2,540 responses (32%) were deemed valid. The mean age of the participants was 23 years. The majority were women (77%), identified as white (70%), and had some experience in higher education (82%). Nearly half of the valid sample (48%) scored ≥6 on the Prodromal Questionnaire (PQ-16).

**Discussion:**

The proportion of participants scoring ≥6 on the PQ-16 is consistent with the findings from other European studies in the general population and in outpatient mental health settings. The procedures and sample characteristics reported here provide context for further analyses using the ENTER tool. In addition, the findings highlight the considerable challenge of fraudulent and inauthentic responses in online research—an issue that may have been amplified by the use of financial incentives and recruitment via social media.

## Introduction

Psychotic disorders, such as schizophrenia, comprise one of the world’s leading causes of health-related disability (1) and are linked to a high personal burden to the affected individuals, their families, and their carers (2). The first episode of psychosis (FEP) typically occurs during adolescence or early adulthood (3) and is often preceded by a prodromal phase characterised by attenuated psychotic symptoms (4). Attention has therefore been directed towards the early detection of individuals meeting the clinical high-risk for psychosis (CHR-P) criteria (5). CHR-P individuals are typically between 14 and 35 years of age and accumulate various environmental, genetic, and individual risk factors for psychosis (6, 7). The CHR-P status is associated with an increased risk of developing a psychotic disorder (OR = 9.32), which peaks at 2–3 years following the initial clinical assessment and continues to gradually increase over time to a cumulative risk of 0.35 at 10 years of follow-up (8, 9).

Effective detection of young people who might benefit from specialised support is one rate-limiting step for improving outcomes through preventive interventions (10). Currently, the detection of CHR-P individuals is dependent on the available material and human resources of services (11). Psychosis risk assessments are commonly performed by trained personnel in help-seeking individuals who have already been referred or have self-referred to specialised CHR-P clinics (12). Evidence suggests that this is highly inefficient (10). A study of a local NHS Trust found that only 5% of individuals who had FEP had been detected by a local CHR-P service (13). In Australia, the Headspace initiative is a frontline youth mental health service (14, 15). However, despite their “one-stop shop” approach, estimates suggest that only 12% of individuals with FEP are detected during the CHR-P phase (16). As such, the preventive potential of CHR-P services remains largely untapped.

The delayed detection of prodromal symptoms is a major contributing factor to negative long-term outcomes in at-risk individuals (17, 18). Therefore, detecting young people in the community who are experiencing prodromal symptoms and signposting them to specialist treatment may be a step towards improving the outcomes of psychosis. This also aligns with UK clinical guidelines (19, 20), which require the prompt detection and treatment of CHR-P individuals. Promising future avenues for improving the screening and detection of CHR-P individuals in the general population are currently under investigation and include, among others, individualised transdiagnostic (21) or poly-environmental (22, 23) risk calculators, computerised assessments (24), and e-detection strategies aimed at the general population. Among these, e-detection approaches can allow for quick and easy implementation of risk enrichment and detection strategies via the use of web-based versions of self-report scales, such as the Prodromal Questionnaire (PQ-16) (25). This can potentially be used as an initial filter to identify individuals presenting with attenuated psychotic symptomatology who would benefit from a specialised assessment.

Online research has many other advantages, such as having a greater reach, not being limited by geographical location like specialised CHR-P clinics, having increased accessibility, and being able to recruit larger sample sizes in less time with fewer resources for both researchers and participants (26). For mental health research in particular, it can help recruit hard-to-reach and low-prevalence populations and can encourage disclosure of sensitive information, such as mental health symptoms, due to the increased anonymity (26, 27). On the other hand, online health research is faced with important challenges that call for proactive mitigation strategies, notably the risk of fraudulent responses that have been found to affect as low as 3% to as high as 94% of participants in studies (28).

The multi-site ENTER (E-DetectioN Tool for Emerging Mental DisoRders) study aimed at testing and refining an e-detection strategy for the identification of individuals at risk of psychosis in the general population. A longitudinal design was employed to investigate the ability of a self-administered screener (ENTER) to detect true CHR-P, as measured by clinical interview assessments. This method builds upon the Youth Mental Health Risk and Resilience Study (YouR-Study), which provided initial evidence for the feasibility of a web-based screening platform to detect CHR-P individuals in the general population (12). Expanding upon the YouR-Study, ENTER collected information on the following: 1) environmental risk and protective factors; 2) prodromal symptoms of bipolar disorder; and 3) cognitive and speech analysis. All factors have been previously associated with increased risk of psychosis. The aim of the ENTER study was to determine which factors from the screener improve the detection of CHR-P.

This paper outlines the ENTER procedure and recruitment method for automatic digital community screening for psychosis risk, as well as the general sociodemographic characteristics and the PQ-16 scores in the resulting self-selected sample. In addition, this paper describes the cleaning process employed for sample validation to ensure the integrity of the final ENTER study sample.

## Methods

### Ethics

For the UK sites, the study had NHS Research Ethics Committee approval, and all participants gave written informed consent for participation. The study was registered on ClinicalTrials.gov (NCT05127278). The Italian site adopted the same protocol, but was funded by a different grant and is therefore not listed on ClinicalTrials.gov. At the Italian site, the study was approved by the Ethical Committee of IRCCS San Matteo (no. 0044135/22).

### UK site recruitment and study promotion

Participants were recruited from the general population in London and Glasgow. A combination of invitation e-mails through universities and colleges, flyers, and social media (e.g., Facebook and Instagram) was employed (see **Supplementary Figure S1**). Potential participants could obtain additional information about the study and a link to initiate the electronic consenting process, which, after completion, directed participants to the online screener section of the ENTER study.

A pilot version of the ENTER website was presented at the Young Person’s Mental Health Advisory Group (YPMHAG) at King’s College London, and feedback from young experts by experience was collected to inform the development of the website and the promotional materials.

The criterion for participant inclusion in the screener was age 12–35 years. Absence of a previous psychotic disorder was assessed at the interview stage. Participants were compensated with a £10 Amazon voucher for completing the 30-min screener. Recruitment started in February 2022 and ended in December 2024.

### Italy site recruitment and study promotion

Recruitment strategies included e-mail invitations sent to university students, dissemination via internal WhatsApp groups of individual faculties, outreach through student associations, and the distribution of posters and brochures in common youth gathering places and general practitioners’ clinics. The study was also promoted via social media platforms (e.g., Instagram and LinkedIn). Participants at the Italy site in Pavia were recruited via e-mail invitations to students at universities in Pavia. In Italy, financial compensation to participants in clinical research is not permitted, except for reimbursement of direct expenses or documented loss of earnings as per the national implementation of EU Regulation no. 536/2014 and the official model approved by the Italian Coordination Centre of Ethics Committees (29). Therefore, unlike in the UK, participants at the Pavia site did not receive financial compensation.

### Study design

#### Screener

The consent form and screener was built and managed through the online platform REDCap (30).

At enrolment, participants provided their age and site location before viewing the participant information sheet (PIS). The PIS explained that, after completion of the screener, eligible participants—the criteria were not disclosed to them—might be contacted by e-mail or phone to schedule an interview. At the bottom of the PIS, participants gave consent and submitted their contact details.

In the screener, participants were asked to complete demographic questions, the prodromal questionnaire (PQ-16) (31), the Psychosis Polyrisk Score (PPS) assessment (23), and the Digit Symbol Substitution Test (DSST) (32). Participants from the London and Italy sites also completed the self-rating version of the Bipolar Prodrome Symptom Scale (BPSS-AS-P) (33). The BPSS-AS-P scale was not included for the Glasgow site.

In the general population, the Cronbach’s alpha for the total score on the PQ-16 has been found to be 0.774 (31).

Participants were then directed to the online experiment builder platform Gorilla (www.gorilla.sc). Here, speech samples were collected using the eight images from the Thematic Apperception Test (TAT) as stimuli (34). When the image appeared, the accompanying text read, “Please describe what you see in this image. Please speak for the full minute—the recording has started,” and at 30 s, “How does this image make you feel?”

Across sites, participants were also asked the following question at the end of the PPS assessment: “How distressing did you find the process of completing this questionnaire?,” with the response options ranging from “not distressing at all” to “extremely distressing.”

**Figure 1:**
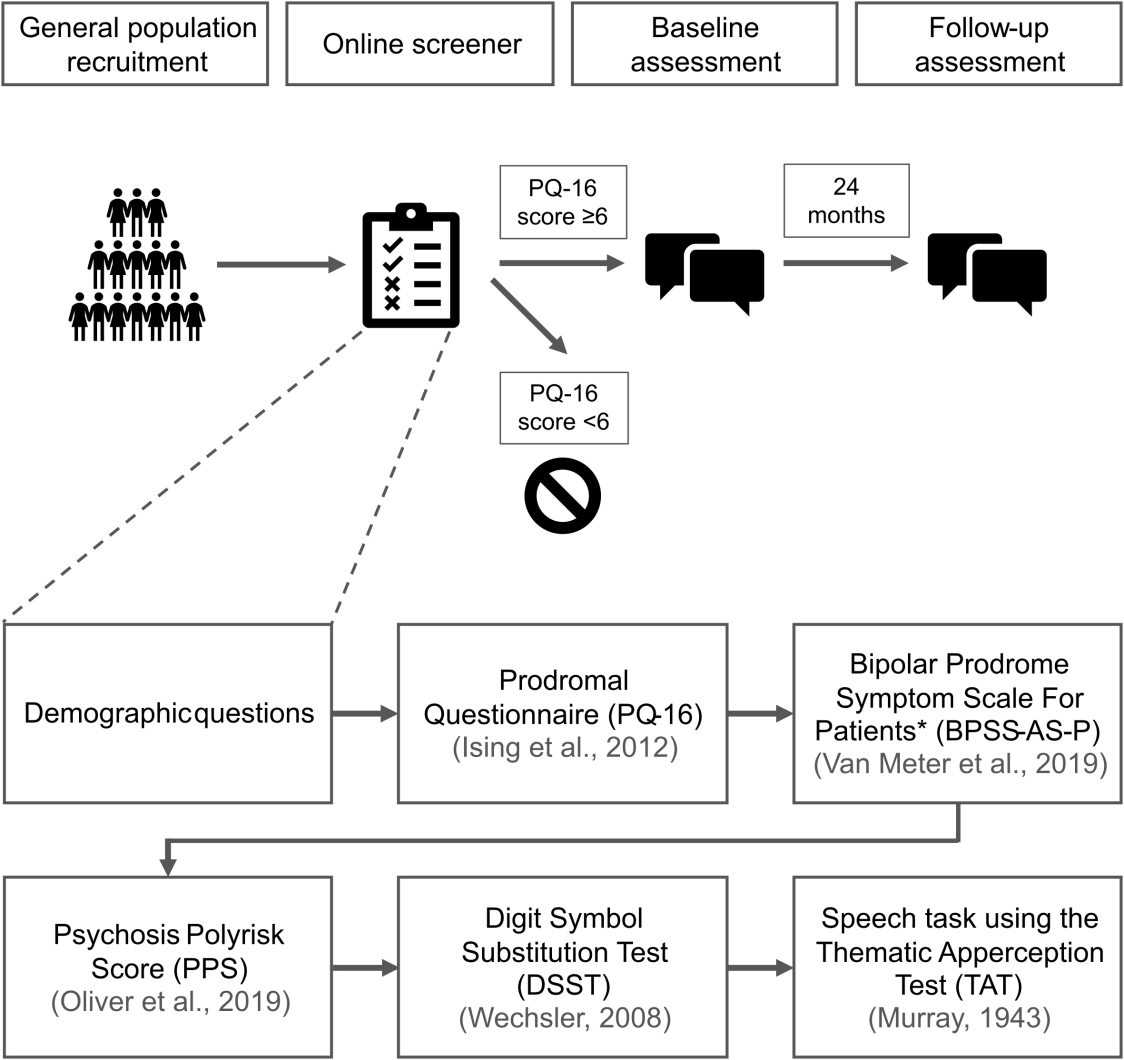
ENTER (E-DetectioN Tool for Emerging Mental DisoRders) study design. *The BPSS-AS-P (Bipolar Prodrome Symptom Scale) was not used at the Glasgow site.

**Figure 1** shows the overall study design.

#### Data cleaning

Given the prevalence of suspected fraudulent or inauthentic responses in web-based survey research, a multilayered data cleaning system was implemented to improve the integrity of the ENTER study. The detailed methodology can be found in the **Supplementary Material**. This manuscript uses the umbrella term “invalid data” to refer to entries that were judged to be potentially fraudulent or inauthentic, as well as lower-quality responses, such as those failing attention checks or not meeting the time thresholds.

### Statistical analysis

Data visualisation and analysis were performed using R Statistical Software (v4.3.1; 35). The Kolmogorov–Smirnov test and the Mann–Whitney *U* test were performed on the PQ-16 data (see **Supplementary Material**).

## Results

### UK data cleaning

After passing CAPTCHA, 8,009 entries across all sites were submitted via the screener. One participant asked to have their data withdrawn.

**Figure 2:**
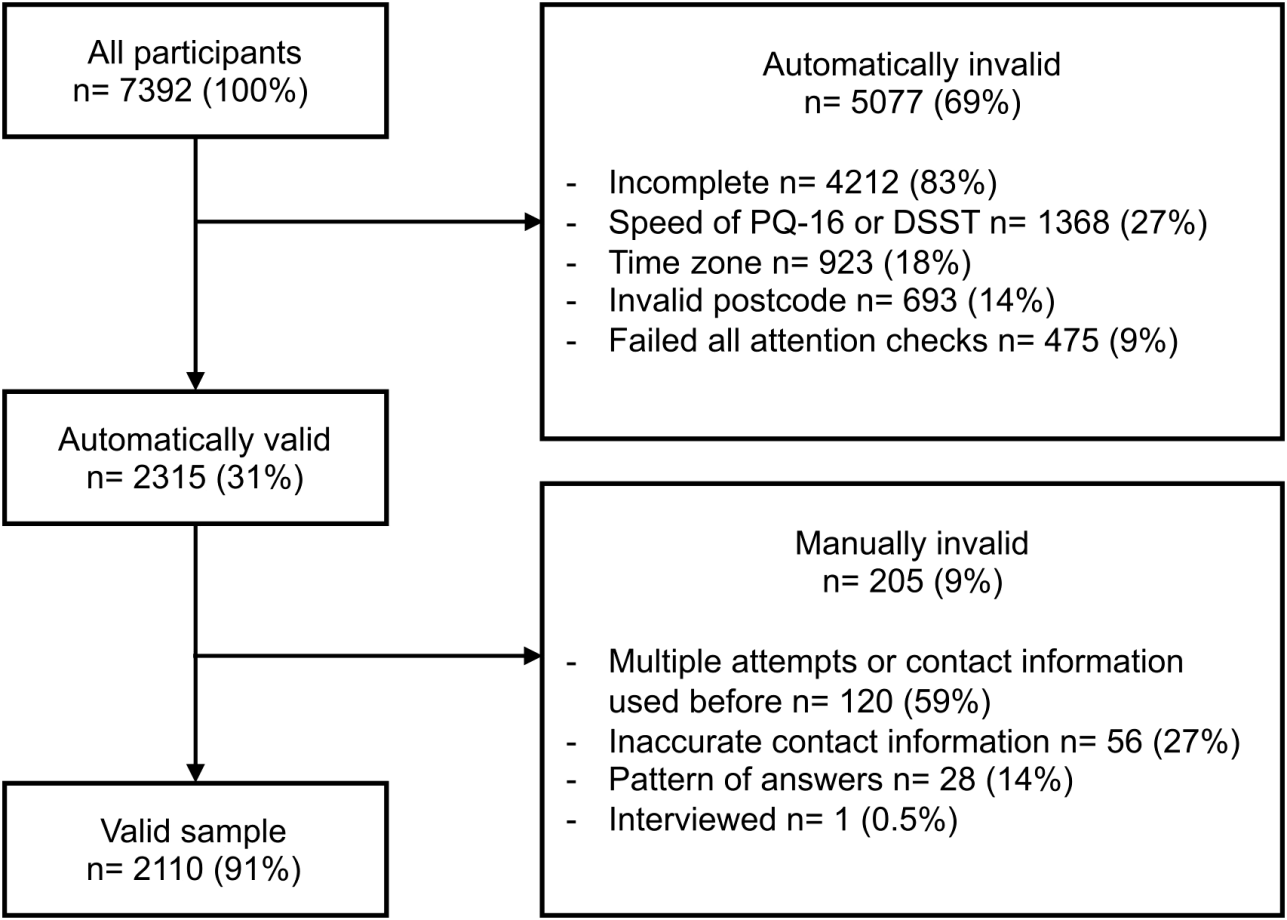
UK validation process with the number of entries excluded at each stage. The entries in the automatically invalid box may fall into multiple categories, making them invalid. In the manually invalid category, the entries were categorised into one reason.

The UK screener received 4,105 entries via the Glasgow site and 3,261 entries via the London site; 26 entries did not reach the question asking where they were recruited from. The ratio of valid to invalid entries was around 2:5 (**Figure 2**) (see **Supplementary Material**). The most common reason for the entries being excluded (83%) was that they were significantly incomplete (i.e., had not finished the DSST). A substantial number of entries (27%) were also ruled out due to completing the PQ-16 or DSST faster than it would be possible to make a considered decision. This may have been due to bots or people speeding through the screener. After data cleaning, the valid UK sample consisted of 2,110 entries.

### Italy data cleaning

**Figure 3:**
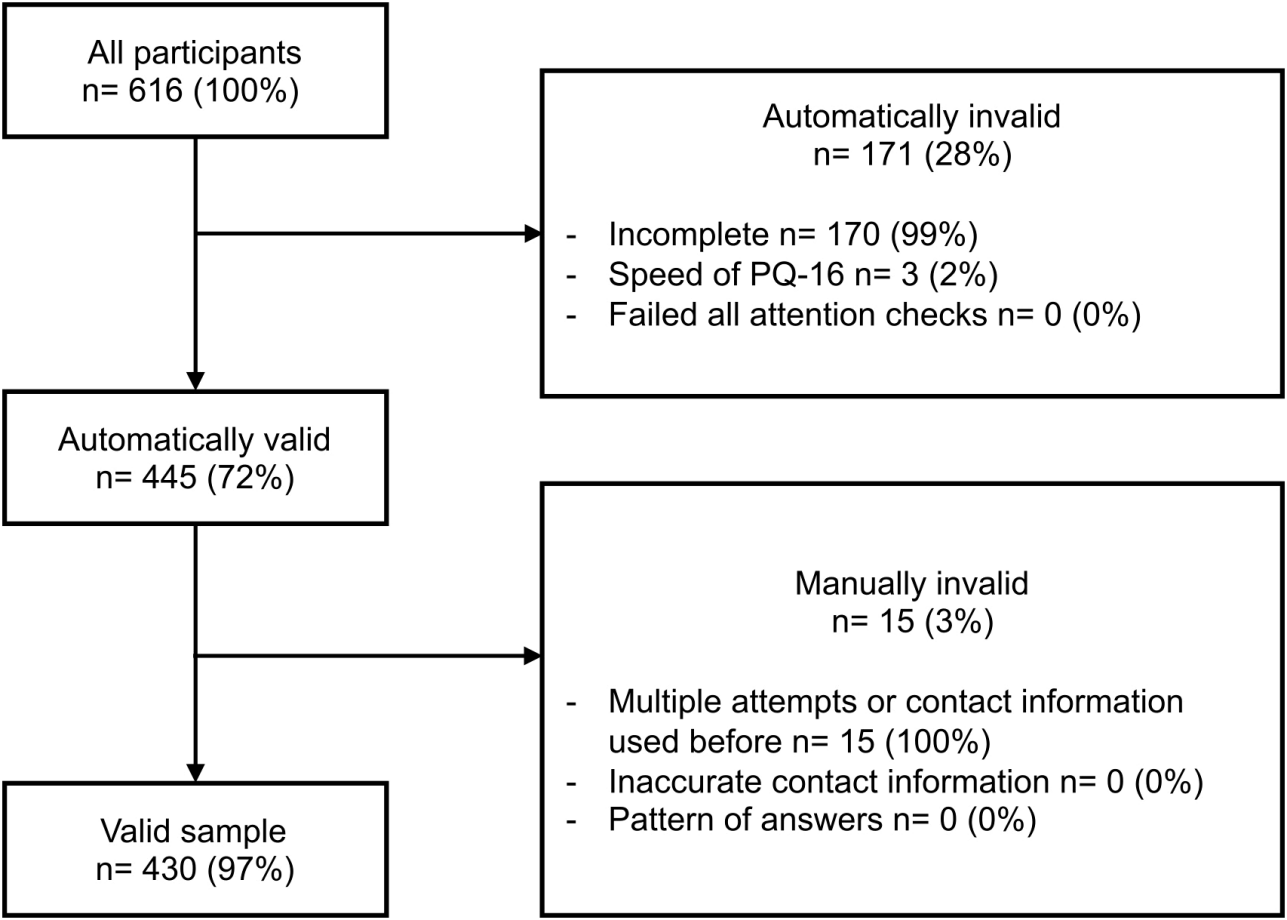
Italy validation process with the number of entries excluded at each stage. The entries in the automatically invalid box may fall into multiple categories, making them invalid. In the manually invalid category, the entries were categorised into one reason.

The Italy screener received 616 entries. Similarly, the most common reason for entries being excluded in the Italy sample was that they were significantly incomplete (99%) (**Figure 3**). The only reason for invalidating automatically valid entries was because the participant had already completed the screener previously (3%). After data cleaning, the valid Italy sample consisted of 430 entries.

### Recruitment of the valid sample

**Figure 4:**
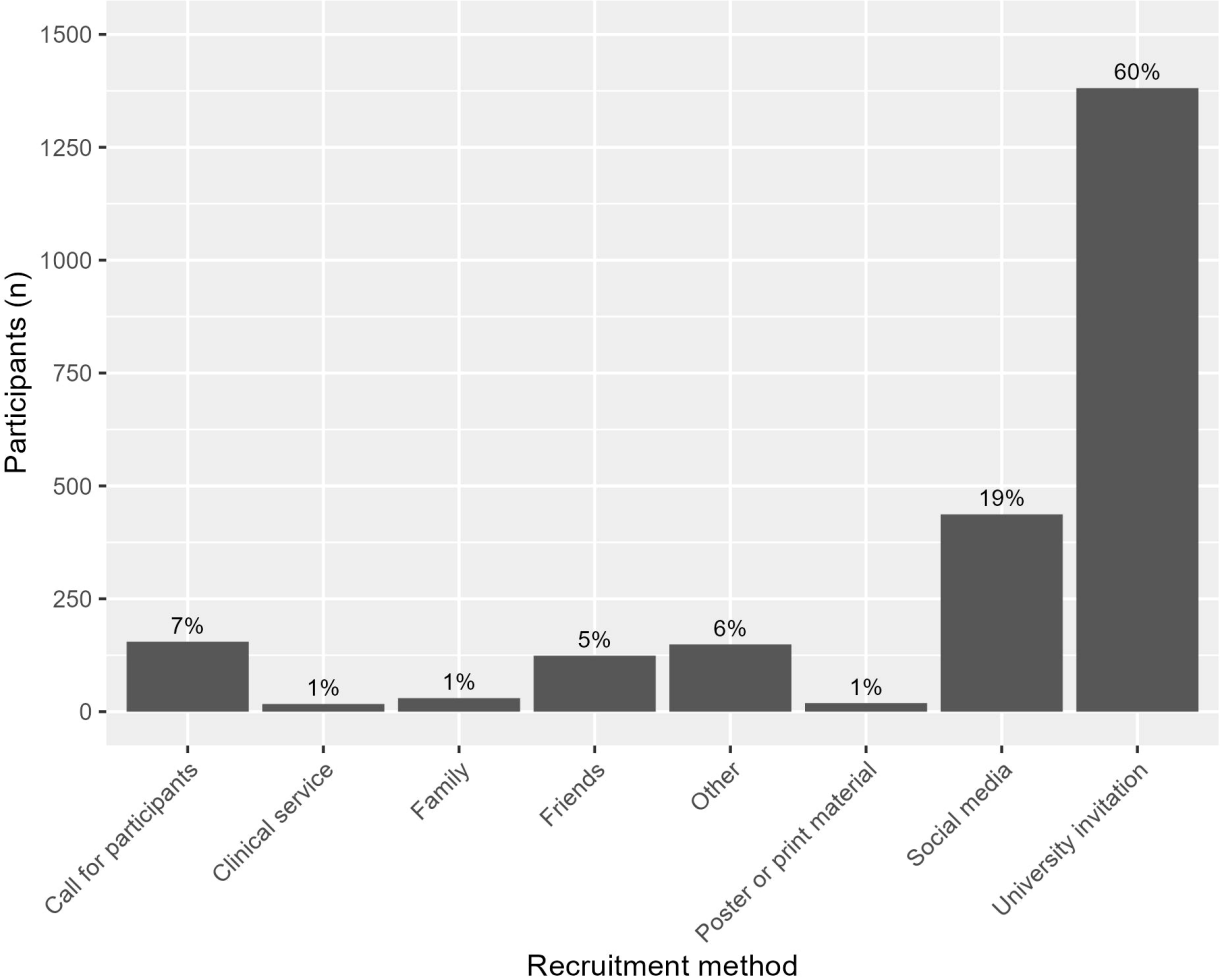
Frequency of the recruitment methods from participants that answered this question (*n* = 2,312).

Participants were predominantly recruited through universities (**Figure 4**). The majority of the Italy sample (83%) did not respond to the recruitment question.

### Sample demographics

The demographic information for the valid sample is summarised in **Table 1**. One participant did not provide a date of birth.

**Table 1:** Demographics of the valid sample.

Characteristic	Total (n = 2,540)	
Age (years)		
Mean (SD)	23 (4.6)	
Min, max	12, 35	
Sex at birth, *n* (%)		
Female	1,958	(77.1)
Male	582	(22.9)
Gender, *n* (%)		
Female	1,819	(71.6)
Male	580	(22.8)
Non-binary	100	(3.9)
Other	15	(0.6)
Prefer not to say	26	(1.0)
Ethnicity (self-assigned), *n* (%)		
Asian	485	(19.1)
Black	71	(2.8)
Mixed	113	(4.4)
Other	94	(3.7)
White	1,774	(69.8)
Prefer not to say	3	(0.1)
Migrant status, *n* (%)		
First-generation migrant (came to the UK/Italy after age 5)	772	(30.4)
First-generation migrant (came to the UK/Italy before age 5)	103	(4.1)
Second-generation migrant (born in the UK/Italy, one or both parents born abroad)	259	(10.2)
Born in UK/Italy and not first- or second-generation migrant	1,406	(55.4)
Education, *n* (%)		
Completed post-graduate qualification	322	(12.7)
Some post-graduate studies	368	(14.5)
Completed graduate/professional qualification	199	(7.8)
Some graduate/professional school	118	(4.6)
Completed college/technical school/undergraduate	378	(14.9)
Some college/technical school/undergraduate	699	(27.5)
Completed high school	393	(15.5)
Some high school	57	(2.2)
Completed primary school	3	(0.1)
No schooling	3	(0.1)
First-degree relatives with psychosis, *n* (%)		
None/unknown	2,441	(96.1)
One	92	(3.6)
Two	7	(0.3)
Tobacco use, *n* (%)		
Non-smoker	1,884	(74.2)
Non-daily smoker	344	(13.5)
Daily smoker	312	(12.3)
Cannabis use, *n* (%)		
Non-cannabis user	1,924	(75.7)
Past cannabis user	328	(12.9)
Current cannabis user	288	(11.3)

### PQ-16 scores

**Figure 5:**
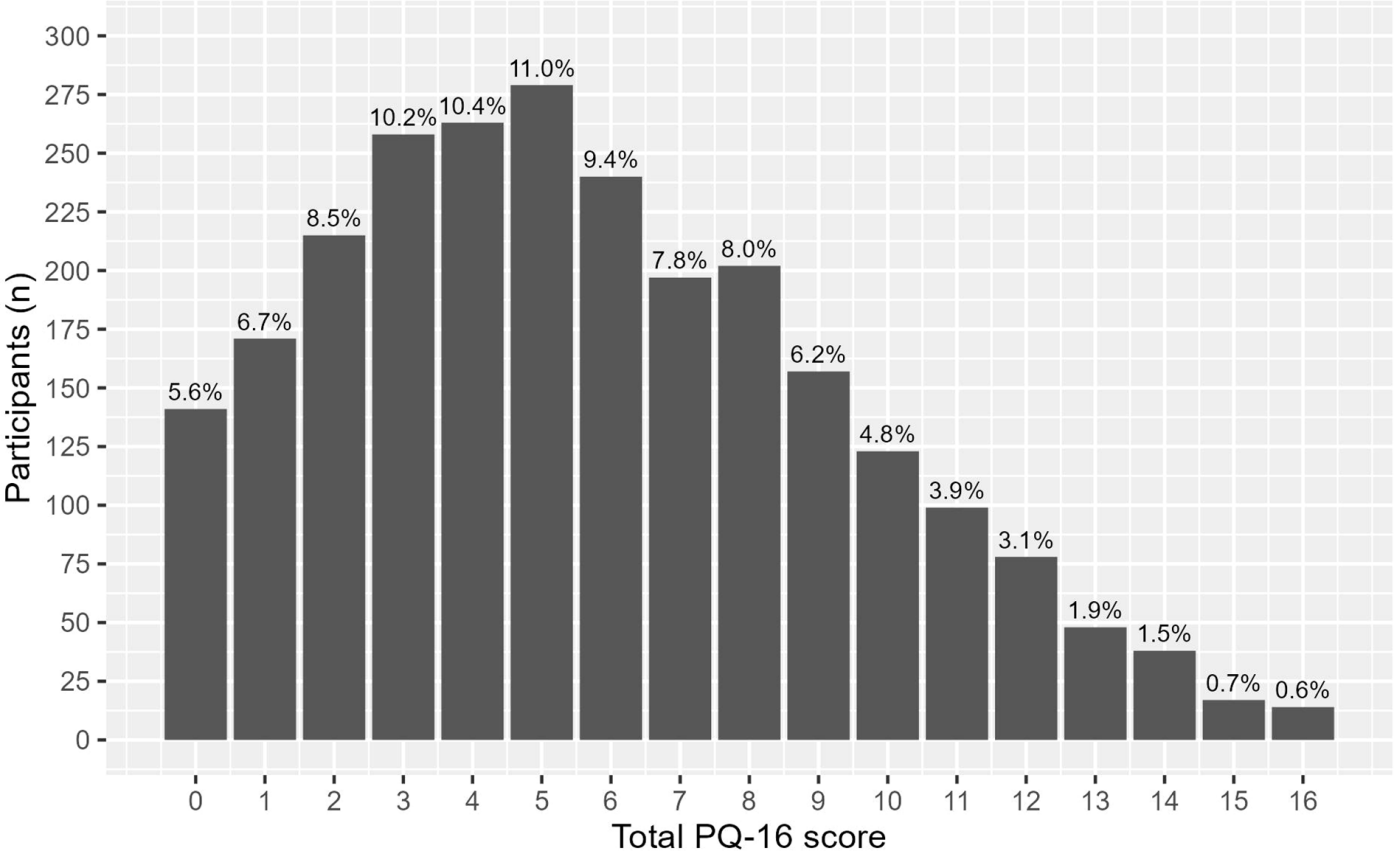
Frequency of the prodromal questionnaire (PQ-16) scores.

The across-site valid sample had a full range of PQ-16 scores, with a median score of 5 (**Figure 5**). The mean score was 5.7 (SD = 3.7). Approximately 48% (*n* = 1,213) reached the threshold (six items) to be invited to the assessment phase. The distress scores ranged from 0 to 43, with a mean of 8.6 (SD = 7.1) and a median of 7.

### Screener experience

Just over half of the sample (53.6%) found that the screener was “not distressing at all.” Of the rest, 43.0% found it “mildly distressing,” 3.1% “very distressing,” and 0.2% “extremely distressing.” The remaining 0.1% did not answer the question.

## Discussion

The aim of the ENTER study was to implement digital screening for emerging psychosis in the community and identify key factors from the screener that improve the detection of true CHR-P as assessed by the CAARMS. The specific aims of this paper were to outline the ENTER procedure, the recruitment methods for automatic digital community screening, and the data cleaning process and to report on the general sociodemographic characteristics and the results of the PQ-16 in the resulting self-selected sample.

The study screened 2,540 participants from sites in London (England), Glasgow (Scotland), and Pavia (Italy). The majority of the sample were of the female sex (77%), self-described white ethnicity (70%), and had some experience in higher education (82%). The mean age was 23 years (the range 12–35 years was set as an inclusion criterion). Just under half (45%) were migrants, either first or second generation. University invitations were the most effective recruitment strategy, accounting for 60% of the participants, which may explain the demographics of the sample.

Almost half of the sample (48%) scored ≥6 on the PQ-16, reaching the threshold set for CAARMS assessment. This was similar to the YouR-study, which found that 52% of its participants aged 16–35 years met the PQ-16 threshold (12). The Tone-P study also employed a web-based screening approach based on the YouR-study to identify CHR-P individuals and found that 43% of participants aged 18–35 years reached the PQ-16 threshold (36).

In addition, the percentage falls within the 35%–65% range reported by other European studies that recruited adolescents and young people from outpatient mental health and child and adolescent services, despite ENTER recruiting from non-clinical sources (37–41). The pretest risk in community samples is typically lower than that in help-seeking samples. The inclusion of many low-risk individuals can create a risk dilution effect, reducing the proportion of true CHR-P cases. To mitigate this and reduce false positives, community samples can be enriched by focusing on participants with a higher pretest risk, for example, those scoring ≥6 on the PQ-16 (42). The rationale for the PQ-16 threshold was based on the finding of Ising et al. (31) that a score of 6 or more on the PQ-16 has a high true-positive rate and specificity when differentiating people at ultra-high risk (UHR) of psychosis from those who are CAARMS-negative. There is some discussion as to whether this threshold is high enough and should take into account the distress scores, especially in non-help-seeking general population samples (37, 39, 41). Subsequent papers on ENTER will publish the CHR-P status of this sample.

This study also highlighted the significant challenge of potentially fraudulent or inauthentic responses in online community samples. Ultimately, in our UK sample, where the participants were paid, over 70% of the responses were deemed invalid and were excluded from the analysis. It is important to note that the main reason for data exclusion was significantly incomplete responses.

Incentive levels may have played a role; for example, ENTER provided a £10 Amazon voucher upon screening completion for the UK sample, while the YouR-study only offered entry into a prize draw. While financial remuneration can help remove barriers to access, it has been found that studies with incentives receive six times more fraudulent behaviour by participants than studies that do not include participant payments (26, 43). The focus of cleaning the data from the Italy sample was on ensuring that the clinical measures were complete and therefore the validation process is not directly comparable (see **Supplementary Material**). However, the Italian sample, which was not financially incentivised, had no participant responses invalidated due to not passing at least one attention check, providing a pattern of responses or inaccurate contact information.

The promotion of the study on social media may have further contributed to invalid responses, as this can attract participants primarily seeking incentives and facilitate the sharing of strategies to bypass validation measures (27, 44). In comparison, the YouR-study recruited through e-mail invitations sent to universities and colleges in Glasgow and Edinburgh and through flyers and posters, while the Tone-P study recruited through the undergraduate mailing lists of French and UK universities and Amazon’s Mechanical Turk. In ENTER, the top reported recruitment methods in the invalid sample in order (excluding those who did not finish the screener) were social media (33%, namely, via Facebook), friends (31%), and alleged university invitations (20%) (see **Supplementary Material**).

Given that the aim of the ENTER study was to identify key factors from the screener that improve CHR-P detection, it was paramount to undergo an extensive data cleaning process for future analyses, particularly with machine learning, to avoid the “garbage in, garbage out” problem. Whilst the detection of CHR-P enriched by an online screener is at the research stage, we encourage others conducting online research to proactively anticipate and implement practical considerations to mitigate the risk of fraudulent responses, in particular if monetary incentives are involved. In our experience, no single measure was single-handedly able to successfully identify and screen out all fraudulent respondents. As suggested in previous studies, a combination of automated and manual measures across the whole life span of the project appears to be the most effective strategy (45).

Overall, the results of this study, thus far, showed that online screening for psychosis risk in the general population is a feasible and promising strategy. In addition, real-world clinical settings may naturally mitigate many of the challenges outlined, as there will be a less intensive social media recruitment drive, no financial incentive, and people will be primarily motivated by help-seeking.

In conclusion, nearly half of the young people recruited from the general population who responded to an advert asking “Are you experiencing mental health difficulties?” reached a score of 6 on the PQ-16, which is consistent with the findings of other European studies. The procedures, data cleaning process, and sample characteristics reported here provide important context for future analyses and publications using the ENTER sample. This paper also contributes to addressing the ongoing challenge of fraudulent and inauthentic responses in online research.

## Data Availability

The raw data supporting the conclusions of this article will be made available by the authors, without undue reservation.
