# Supplementary material for: The ENTER study (E-DetectioN Tool for Emerging Mental DisoRders): general population recruitment and data integrity in online screening for psychosis risk
Source: Front Psychiatry. 2025 Nov 18;16:1665854. doi: 10.3389/fpsyt.2025.1665854 (PMC12670249; doi:10.3389/fpsyt.2025.1665854)
Supplement: Supplementary file 1 [file Supplementaryfile1.docx]

**Supplementary material**

Contents

1. Advertisement material
2. Prevention and detection of potentially fraudulent or inauthentic responses

2.1 Real-time detection of potentially fraudulent and inauthentic responses in the UK sample

2.2 Automated post hoc identification of potentially fraudulent and inauthentic responses in the UK sample

2.3 Manual post hoc identification of potentially fraudulent and inauthentic responses in the UK sample

2.4 Detection of potentially fraudulent and inauthentic responses

2.5 Statistical analysis

1. Potentially fraudulent activity and low-quality responses in the sample

3.1 Post-hoc decisions

3.2 Observations

1. Discussion
   1. Limitations
2. References
3. ***Advertisement material***


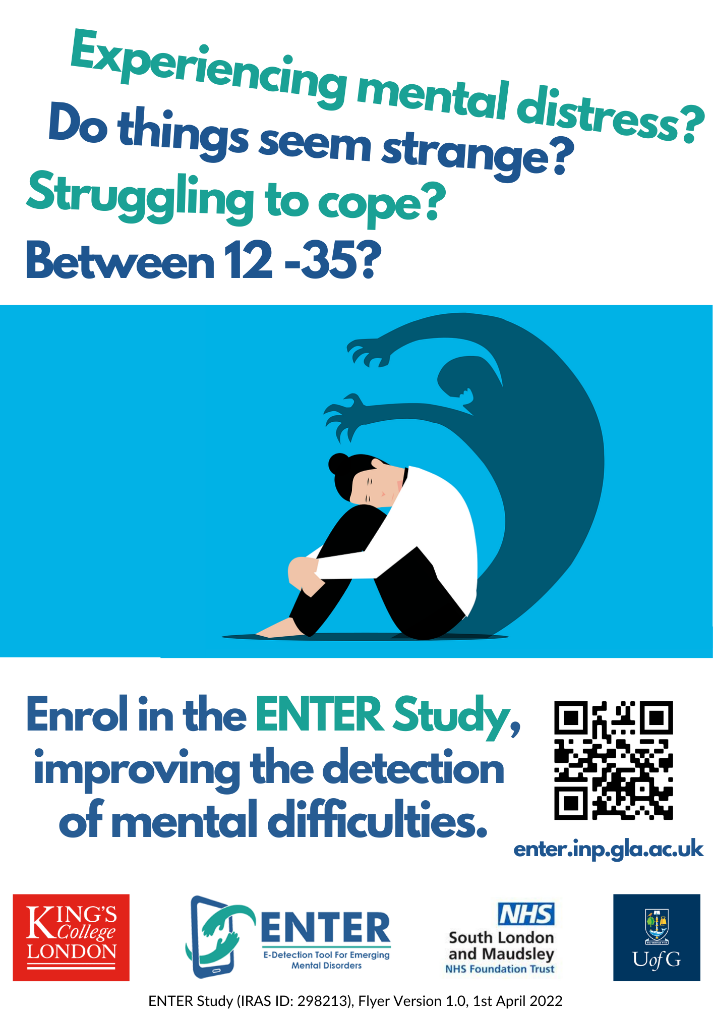


*Supplementary figure 1- ENTER flyer*

***2. Prevention and detection of potentially fraudulent or inauthentic responses***

Given the prevalence of fraudulent responses in web-based survey research, a multi-layered system was implemented to improve the integrity of the ENTER study. As indicated by guidelines and previous experiences in online research, no single security measure is entirely effective at detecting and deterring fraudulent responses (Bonett et al., 2024). As such, the ENTER study implemented a combination of automated and manual validation procedures, as described below.

*2.1 Real-time detection of potentially fraudulent and inauthentic responses in the UK sample*

During the online screener stage, the following automated checks were implemented in August 2022 to detect potentially fraudulent and inauthentic responses in real-time in REDCap (Supplementary figure 2):

1. Computer Automated Public Turing test to tell Computers and Humans Apart (CAPTCHA) prior to the online consent process and again before the screener;
2. the current postcode not matching the standard UK format;
3. completion of online screener from time zones outside of UTC and UTC+1;
4. unusually short completion times for the PQ-16 and DSST

If any CAPTCHA was failed or criteria 2-4 were met, the participant was classified as invalid and their participation in the study was automatically terminated.

*2.2 Automated post hoc identification of potentially fraudulent and inauthentic responses in the UK sample*

If a participant passed the real-time automated checks, and provided a complete dataset of responses, their data was then evaluated via a series of automated post hoc validations. This process included the following automated checks, some of which overlap with the checks previously described (Supplementary figure 2):

1. failure to correctly respond to attention questions which were distributed across the survey;
2. completion of “honeypot” question hidden from participants’ view;
3. completion of online screener outside of the UTC and UCT+1 as indicated by the device’s time zone at the time of completion of the survey (registered in Gorilla);
4. incomplete dataset, as indicated by failure to at least partially complete the final speech sample acquisition task (Gorilla).

Responses that passed the automated checks previously described were preliminarily considered to be valid. However, a participant meeting any of the following criteria was classified as “suspicious”:

1. the residential address, phone number or email address had already been reported by another participant;
2. partially complete speech sample acquisition task.

*2.3 Manual post hoc identification of potentially fraudulent and inauthentic responses in the UK sample*

Finally, records preliminarily considered to be valid were manually assessed on a one-to-one basis. The process was conducted by trained researchers of the ENTER study. When in doubt regarding the validity of a certain record, a consensus was reached by a collaborative discussion between the staff and senior researchers. The manual validation process considered the following information when reaching a conclusion about the validity of responses:

1. valid UK residential address;
2. non-duplicated and valid phone number and email address. When a duplicated attempt was detected, only the first complete dataset for that participant was considered valid, and subsequent attempts were rejected.

Other criteria used during the manual validation process included:

1. identification of automated rapid survey submission attempts via the survey start time and response patterns (e.g., total PQ-16 scores);
2. a mismatch in reported age across two separate age checks in the online screener (i.e., during consent process and PPS instrument).

If doubts persisted regarding the validity of a particular participant:

1. natural speech samples collected via Gorilla were assessed to confirm the survey was completed by a real person.


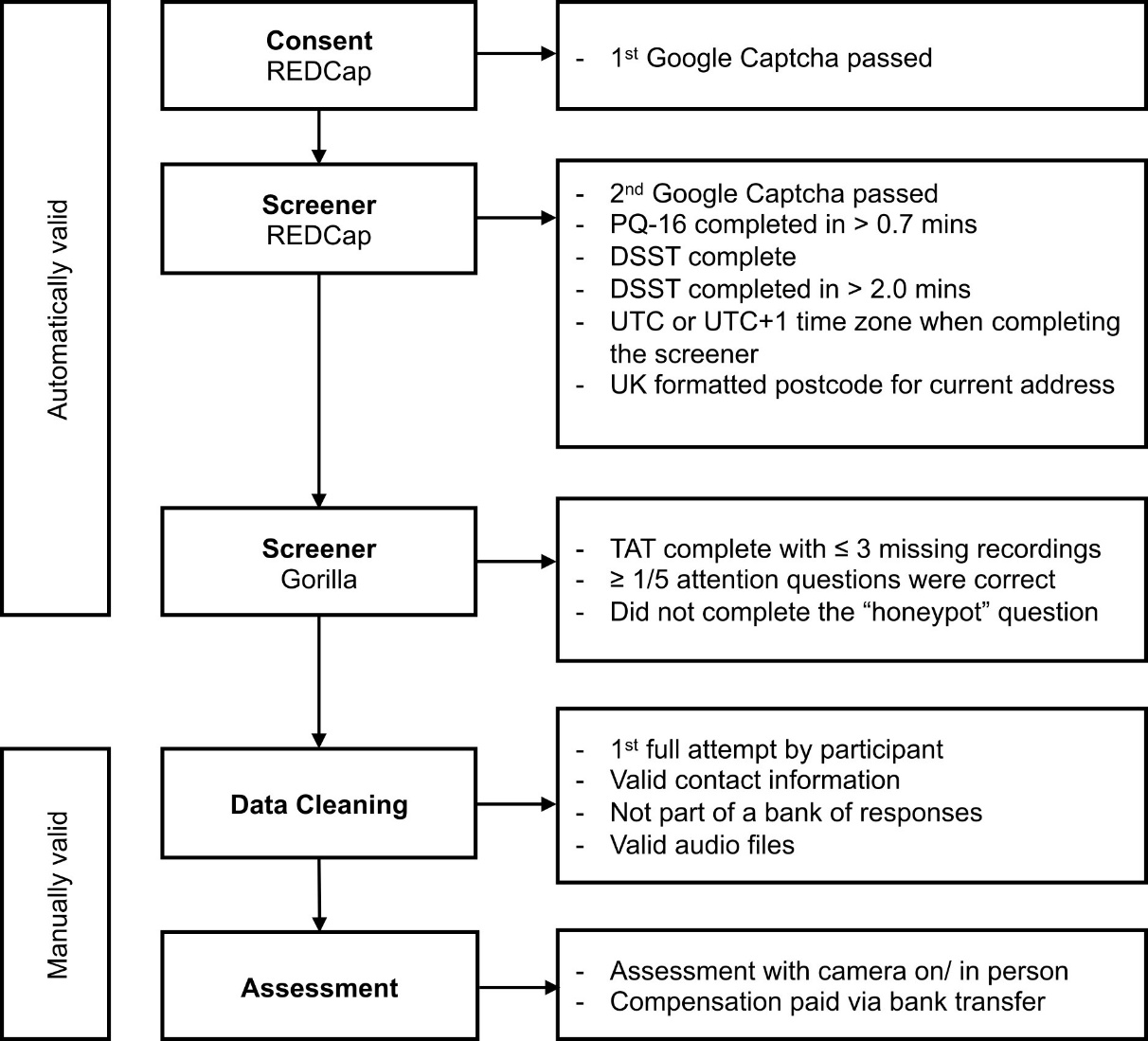


*Supplementary figure 2- The validation process in the UK samples*

*2.4* *Detection of potentially fraudulent and inauthentic responses in the Italy sample*

The participants from the Italian site were not paid for their participation. The validation of the Italy sample was focused on clinical measures (Supplementary figure 3).


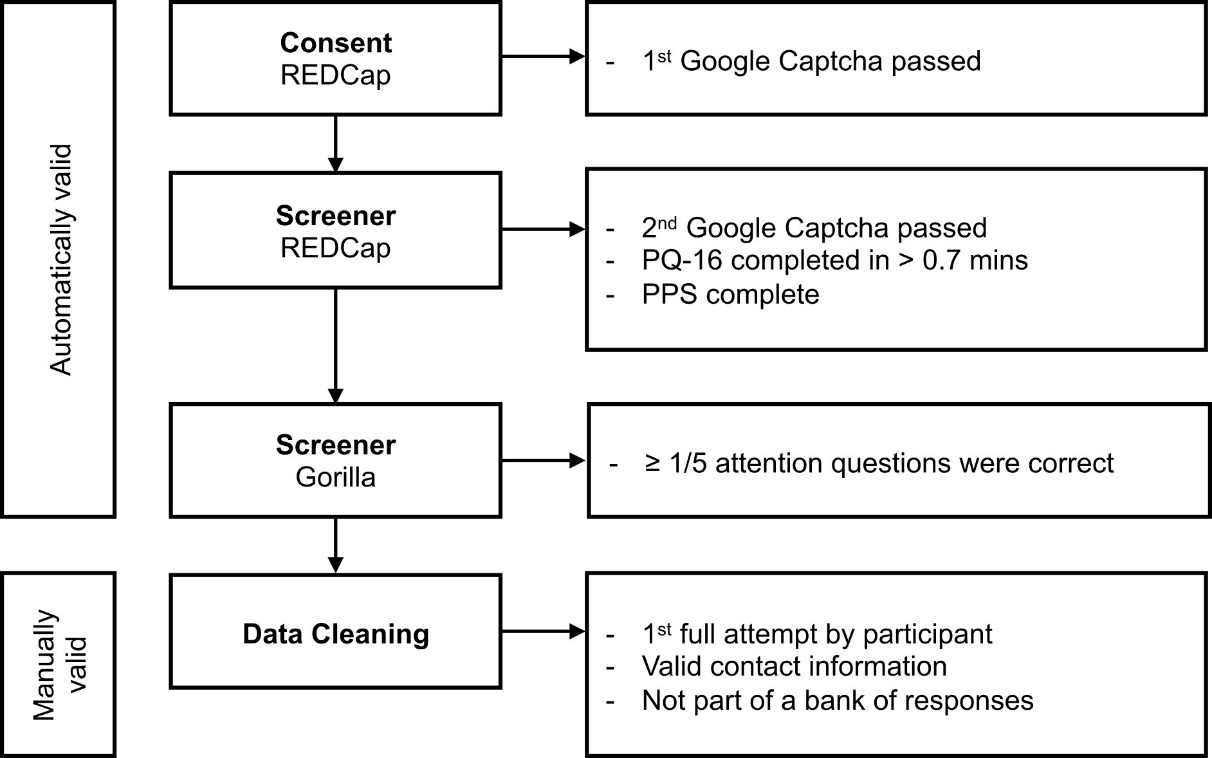


*Supplementary figure 3- The validation process for the Italy sample*

*2.5 Statistical analysis:*

Analysis was performed using R Statistical Software (v4.3.1; R Core Team 2023). The Kolmogorov-Smirnov test and Mann Whitney U test were performed on the PQ-16 data.

***3. Potentially fraudulent activity and low-quality responses in the sample***

*3.1 Post-hoc decisions*

**Postcodes**: When finding repeated postcodes, it was taken into consideration that many students live together or in student accommodation. Additionally, if people gave a postcode outside of London or Glasgow (the two recruitment areas), this was not considered invalid as this may be a student's home address as opposed to a term-time address unless there were additional suspicious factors (see Supplementary table 1). Sometimes, entries gave a landmark for their current postcode, such as Buckingham Palace, which may have been to protect their privacy. However, only entries with obvious privacy concerns such as "no", "na", "not comfortable providing", "prefer not to say" and "would rather not say" were validated. Where half a postcode was given, entries were validated unless additional suspicious factors existed.

**Age**: As a rule, entries where age mismatched the DOB by ≤1 year were validated to allow for people who had rounded up or down their age unless there were additional suspicious factors.

*3.2 Observations*

**Responses**: The response characteristics for supposed fraudulent profiles are shown in Supplementary table 1.

When analysing the invalid entries, both those screened out automatically and manually were included, but those who did not finish (voluntarily or due to real-time detection) were excluded, resulting in a sample of n = 755. The notable demographic differences between this invalid and the valid UK sample were sex and ethnicity. The invalid sample was notably more male (60% vs 23%) and had substantially more entries that identified their ethnicity as black (44% vs 3%). In addition, the invalid sample was more likely to mark that they had a relative with psychosis (28% vs 4%) and five entries reported having three relatives with psychosis. In contrast, the highest number of relatives with psychosis in the valid sample was two.

The average PQ-16 score of the invalid sample was 6.5 (SD = 4.9), which was significantly higher than that of the valid sample (p = .013). The median PQ-16 score of this group was 6. Additionally, the distress scores, which ranged from 0 to 46, were significantly higher than that of the valid sample (p = .006), with an average score of 10.5 (SD = 9.2) and a median score of 8.

ENTER did not recruit via TV and radio or Youtube. In the valid sample, 0.5% answered yes to one of these non-existent recruitment methods, compared to 3.8% of the invalid sample. The top reported recruitment methods in the invalid sample in order were social media (namely Facebook), friends and university invitation.

**Honey pot question:** The question that was hidden from participants visually but could be read by a computer (and therefore bots) asked the participant to pick a number [1-3] from a drop-down list. Only two records completed the honeypot question. These records were automatically rejected for not completing the screener before needing to be rejected because of the honey pot question.

**Payment:** Not automatically sending vouchers prevented the study from having to terminate early. Additionally, having one person in charge of paying vouchers via email for each section of the study made it easier to spot patterns and repeated names (see Supplementary table 1).

**Communication:** When emailing invalid participants to tell them we were not providing payment for their participation, most did not respond. Those who did were directed to senior researchers to discuss further or invited to do the study again.

*Supplementary table 1- Profile of suspected fraudulent or inauthentic participants*

| Data Category | Variable(s) | Response characteristic or pattern |
| --- | --- | --- |
| Personal details | Names | Surname and first name the other way around |
|  | Postcode | Non-residential address (e.g. office/ companies/ gyms), London landmarks, no longer exists, not a real postcode, half a postcode |
|  | Email address | Name not matching the email address, patterns such as a double letter on the end i.e. John Doe, would be [john.doee@email.com](mailto:john.doee@email.com) or a string of numbers that are seen repeatedly on other emails [john.doee924678@email.com](mailto:john.doee924678@email.com) and jane.doe924678@email.com |
|  | Phone number | Landline (sometimes belonging to a company), not a phone number |
|  | Same person based on personal details given but using a different email (e.g. university/ work and personal) or phone number. | |
| Demographics | Age | The reported age not matching the DOB given |
| Response patterns across participants | Bulk responses and patterns | A significant influx of entries at the same time as entries at similar times with matching patterns e.g. same age and half a postcode |
|  | Linking | Postcodes are used by other invalid entries or emails already used with invalid postcodes. |
|  | Emailing | Batches of emails with similar or identical messages sent simultaneously asking for payment.  Emails indicating survey source before disseminating the survey from that source (i.e., emailing the email account used for a linked follow-up component that the participant had not been invited to). |

***4. Discussion***

The invalid sample had higher PQ-16 scores than the validated sample and higher rates of relatives with psychosis than the valid sample. Additionally, there were significantly higher distress scores in the invalid sample. We can cautiously speculate that these findings may indicate experience inflation or that some participants were attempting to provide responses they believed would qualify them for the study or had learned were necessary to advance to the paid assessment stage (Chandler and Paolacci, 2017; Saberi, 2020). Evidence to support this is that some participants with flagged invalid entries emailed an account used only for the linked follow-up component when they had not been invited to this section and would have no way of knowing the account existed unless a previous entry had reached this point. The finding of higher scores was also reported in participants suspected of being fraudulent or inauthentic in Davies et al. (2024). Our fraudulent profiles (as detailed in Supplementary table 1) have considerable overlap with those in Davies et al. (2024).

It was essential to use a combination of methods to screen out different types of invalid entries, such as suspected bots, imposter and or fraudulent participants and entries of low quality (e.g. failing attention checks or not meeting the timing threshold). However, it is acknowledged that even with the measures used, people with adequate resources could evade them, particularly those who use "brute force", where they repeatedly test responses to determine an eligible response (Wang et al., 2020). As people committing fraud continuously update their approaches, human oversight and monitoring are crucial, as well as staying up to date on screening techniques when designing a study (Wang et al., 2020).

However, when designing a study, it is important to balance the participants’ right to privacy with data quality. In the early stages, patient and public involvement (PPI) can play a vital role in ensuring that care is being taken not to add extra burden on participants, increase fatigue or challenge their genuine experiences (Davies et al., 2024; Lawlor et al., 2021). This is even more important when working with potentially marginalised and stigmatised participants (Mistry et al., 2024). Further recommendations can be found in Davies et al. (2024), Lawlor et al. (2021), Mistry et al. (2024), Teitcher et al. (2015) and Wang et al. (2020).

*4.1 Limitations*

With the benefit of insights gained from recent literature on fraudulent participation in online research, we have identified several measures that we would implement in future studies to mitigate this issue. For the ENTER study, participants could have been reimbursed either via direct bank transfer (BACS) or through vouchers redeemable only within the country of the study site where they completed the screener (Mistry et al., 2024).

We chose not to use REDCap’s anonymised Internet Protocol (IP) address logging feature, as the study targeted young people, many of whom were recruited through universities and colleges. In such settings, multiple participants may share the same devices or institutional IP addresses, reducing the utility of IP tracking. Similarly, cookies were not employed, as they can be easily deleted (Teitcher et al., 2015; Wang et al., 2020).

In retrospect, the screening process could have been strengthened by incorporating additional measures such as blocking virtual private networks (VPNs), including open-ended questions, and conducting consistency checks to detect potential fraudulent responses (Davies et al., 2024; Lawlor et al., 2021; Teitcher et al., 2015; Wang et al., 2020).

1. ***References***

Bonett, S., Lin, W., Sexton Topper, P., Wolfe, J., Golinkoff, J., Deshpande, A., Villarruel, A., Bauermeister, J., 2024. Assessing and Improving Data Integrity in Web-Based Surveys: Comparison of Fraud Detection Systems in a COVID-19 Study. JMIR Form Res 8, e47091. https://doi.org/10.2196/47091

Chandler, J.J., Paolacci, G., 2017. Lie for a Dime: When Most Prescreening Responses Are Honest but Most Study Participants Are Impostors. Soc. Psychol. Personal. Sci. 8, 500–508. https://doi.org/10.1177/194855061769820

Davies, M.R., Monssen, D., Sharpe, H., Allen, K.L., Simms, B., Goldsmith, K.A., Byford, S., Lawrence, V., Schmidt, U., 2024. Management of fraudulent participants in online research: Practical recommendations from a randomized controlled feasibility trial. Int. J. Eat. Disord. 57, 1311–1321. https://doi.org/10.1002/eat.24085

Lawlor, J., Thomas, C., Guhin, A.T., Kenyon, K., Lerner, M.D., Drahota, A., 2021. Suspicious and fraudulent online survey participation: Introducing the REAL framework. Methodol. Innov. 14, 20597991211050467. https://doi.org/10.1177/20597991211050467

Mistry, K., Merrick, S., Cabecinha, M., Daniels, S., Ragan, J., Epstein, M., Lever, L., Venables, Z.C., Levell, N.J., 2024. Fraudulent Participation in Online Qualitative Studies: Practical Recommendations on an Emerging Phenomenon. Qual. Health Res. 10497323241288181. https://doi.org/10.1177/10497323241288181

Saberi, P., 2020. Research in the Time of Coronavirus: Continuing Ongoing Studies in the Midst of the COVID-19 Pandemic. AIDS Behav. 24, 2232–2235. https://doi.org/10.1007/s10461-020-02868-4

Teitcher, J.E.F., Bockting, W.O., Bauermeister, J.A., Hoefer, C.J., Miner, M.H., Klitzman, R.L., 2015. Detecting, Preventing, and Responding to “Fraudsters” in Internet Research: Ethics and Tradeoffs. J. Law. Med. Ethics 43, 116–133. https://doi.org/10.1111/jlme.12200

Wang, K., Zhao, Y., Tan, S., Zhang, J., Li, D., Chen, J., Zhang, L., Yu, X., Zhao, D., Cheung, E.F.C., Turetsky, B.I., Gur, R.C., Chan, R.C.K., 2020. Semantic processing event-related potential features in patients with schizophrenia and bipolar disorder. PsyCh J. 9, 247–257. https://doi.org/10.1002/pchj.321
